# Availability of supplies and motivations for accessing voluntary HIV counseling and testing services in Blantyre, Malawi

**DOI:** 10.1186/1472-6963-8-17

**Published:** 2008-01-23

**Authors:** Bwanali H Jereni, Adamson S Muula

**Affiliations:** 1 College of Medicine, University of Malawi, Blantyre, Malawi; 2 Department of Community Health, University of Malawi, College of Medicine, Blantyre, Malawi

## Abstract

**Background:**

HIV counseling and testing is an important intervention in the prevention, control and management of the human immunodeficiency virus (HIV). Counseling and testing can be an entry point for prevention, care and support. Knowledge of the quality of services and motivations for testing by individuals is important for effective understanding of the testing environment.

**Methods:**

A cross sectional explorative study of clients accessing HIV voluntary counseling and testing (VCT) and counselors was conducted in 6 government health centers in Blantyre City, Malawi. We aimed to assess the availability of critical clinic supplies and identify the motivations of clients seeking counseling and testing services. We also aimed to identify the health professional cadres that were providing VCT in Blantyre city.

**Results:**

102 VCT clients and 26 VCT counselors were interviewed. Among the VCT clients, 74% were <=29 years, 58.8% were females and only 7% reported no formal education. 42.2% were single, 45.1% married, 8.8% widowed and 3.9% divorced or separated. The primary reasons for seeking HIV counseling and testing were: recent knowledge about HIV (31.4%), current illness (22.5%), self-assessment of own behavior as risky (15.5%), suspecting sexual partner's infidelity (13.7%) and seeking HIV confirmatory test (9.8%) and other reasons (6.9%). Of the 26 VCT counselors, 14 were lay volunteers, 7 health surveillance assistants and 5 nurses. All except one had been trained specifically for HIV counseling and testing. All 6 facilities were conducting rapid HIV testing with same day test results provided to clients. Most of the supplies were considered adequate for testing.

**Conclusion:**

HIV counseling and testing facilities were available in Blantyre city in all the six public health facilities assessed. The majority of counseling and testing clients were motivated by perceptions of being at risk of HIV infection. In a country with 12% of individuals 15 to 49 years infected, there is need to encourage testing among population groups that may not perceive themselves to be at risk of infection.

## Background

Sub-Saharan Africa has been heavily affected by the Human immunodeficiency virus infection (HIV) and AIDS pandemic. In Malawi adult HIV prevalence is estimated at about 12% [1]. Blantyre, with an adult HIV infection prevalence estimated at 22.3% is among the most severely affected districts in the country [2]. The country is now scaling-up access to antiretroviral therapy such that by March 2007, at least 95,000 individuals were accessing free antiretroviral treatment both from the public and private health sectors. In 2003 it was estimated that 150,000 individuals in Malawi were in need of antiretroviral therapy [3].

HIV voluntary counseling and testing (VCT) has been described as an important intervention for HIV prevention as it may serve as an entry point for prevention and care and support for those found infected [4,5]. The literature has reported on assessments of the acceptability of VCT among different population groups such as pregnant women, tuberculosis patients and the general community [6,7].

In order to inform the effective delivery of VCT services in a country it is important to assess the reasons why individuals present themselves to VCT centers for HIV testing. Killewo et al [8] for instance surveyed rural residents in Kagera, Tanzania to assess their motivations for HIV testing. About 96% who accepted testing reported need to know their HIV status as motivation for choosing HIV testing. Individuals who refused testing suggested that their perceived themselves to be at low risk of infection. In the study by Zachariah et al [9] in rural Malawi, about 50% who consented to HIV testing were motivated through recent knowledge about HIV.

We are unaware of any similar studies on the motivations for HIV testing in an urban area like Blantyre city. We are also not aware on any studies that have reported on the health professional categories offering VCT in Blantyre and the availability of supplies in the testing clinics. We believe that an understanding of the context in terms of staffing and the physical space of HIV counseling and testing can also inform us on the expected quality of care from these testing centers. In March 2005 we conducted a study in an attempt to answer the following questions: a) what are the socio-demographic characteristics of patients seeking VCT services in government health clinics in Blantyre city; b) what is the availability of supplies required for VCT services in these clinics? Also we assessed what were the motivations for seeking HIV testing among clients who presented themselves for testing. We also aimed to identify the cadres of health professionals providing VCT services and assess their work situations.

## Methods

### Study setting

The study was conducted in six public health centers in Blantyre urban, Malawi. Blantyre is the largest commercial city in Malawi with a population of about 750,000. At the time of the survey, health care services were mainly provided by public institutions comprising health centers and the Queen Elizabeth Central Hospital (QECH), a large teaching hospital. The private sector provided a growing but still limited health care services. Voluntary counseling and testing was being provided free of charge to clients in public health facilities. Since our study was aimed to assess care in community-based health centers, the QECH which is a large teaching hospital was excluded.

### Study Design

This was a cross sectional study utilizing an interview questionnaire using both closed and open-ended questions. A checklist was used for the verification of availability of clinical supplies.

### Recruitment of study participants

Study participants comprised HIV test counselors and clients seeking VCT services. Each health center was visited for one to three days (depending on the flow of clients). All counselors available on the days of the visit were invited to participate in the study. A total of 15 to 20 consecutive VCT clients were recruited for the survey at each health center. Clients were recruited by BHJ before attending the HIV counseling session. Data collection was conducted over a period of one month period in 2005.

### Data collection

For the client survey, data were collected on demographic variables and reasons for seeking VCT services. For the VCT provider survey, data were obtained on professional cadre category (nurse, health surveillance, lay person), gender, opening times and days (to assess temporal accessibility), whether the counselor had been trained in counseling, had supervision and availability of supplies at the center. A total of 102 VCT clients were interviewed. All interviews were conducted by BHJ at a place away from hearing range of counselor or any other VCT clients. Response rate was 100%. We assessed the mean number of clients counseled only or counseled and tested per site by reviewing the site record. Observation for confirmation of reports by counselors was used to validate information obtained on the availability of supplies.

### Data analysis

Quantitative data were entered into Excel and frequencies obtained. We also carried also Cross tabulations to assess if there were any differences in responses between males and females. A p value of <0.05 was considered statistically significant. Responses to open-ended questions were categorized based on the main theme of the response.

### Ethical considerations

Institutional ethical review of the study protocol was obtained from the Malawi College of Medicine Research and Ethics Committee (COMREC). Permission to conduct the study was obtained from the Blantyre District Health Officer. All study participants provided individual informed consent which was witnessed by BHJ. Consent was documented for each of the study participant. All study participants received a soft drink after the interview.

## Results

All the six urban public health centers (Bangwe, Chilomoni, Limbe, Ndirande, South Lunzu and Zingwangwa) were providing VCT in March 2005. Each of the health centers was located within a radius of 5 to 8 kilometers from the next health center. 26 counselors and 102 VCT clients were interviewed. The mean number of clients counseled each day per counselor was 8, range 5–12. All the counseling sites were open between Monday and Friday for 7 hours each day. There were no services over the weekend.

### Demographic characteristics of VCT clients

Of the 102 clients seeking VCT during the study period, 51% were in the age range 20 to 29 years, and the majority (50%) had attained secondary education. 41.2% were males while 58.8% were females (p < 0.01). Most were married 45.1% followed by single clients (42.2%). Further description of socio-demographic characteristics is presented in Table 1.

**Table 1:** Socio-demographic characteristics of clients presenting for VCT in government health centers in Blantyre, Malawi, 2005

**Characteristic**	**Frequency (%)**
Age in years	
10 to 19	22 (21.5)
20 to 29	52 (51.0)
30 to 39	15 (14.7)
50 to 49	11 (10.8)
≥50	2 (2.0)
	
**Sex**	
Male	42 (41.2)
Female	60 (58.8)
	
**Education Level**	
None	7 (6.9)
Primary	33 (32.4)
Secondary	51 (50.0)
Tertiary	11 (10.7)
	
**Formal Employment**	
None	63 (61.8)
Yes	39 (38.2)
	
Marital Status	
Single	43 (42.2)
Married	46 (45.1)
Widowed	9 (8.8)
Separated/Divorced	4 (3.9)
	
**Total**	**102 (100)**

### Motivations for seeking HIV testing services

We also aimed to determine the primary (most important) reason for seeking VCT services. The results are presented in Table 2 having been disaggregated by gender. P-values have also been presented with a value of <0.05 as statistically significant. As can be seen in Table 2, males and females did not differ in the various characteristics except one i.e. in having suspected infidelity in a sexual partner. Females were more likely to be motivated of suspicion of infidelity in a sexual partner than males, p < 0.01.

**Table 2:** Primary Reasons for Seeking HIV Counseling and Testing

**Reason for seeking counseling and testing**	**Total (%)**	**Males (% among males)**	**Females (% among females)**	**P values**
Recent knowledge about HIV	32 (31.4)	17 (40.5)	15 (25.0)	0.10
Feeling unwell/illness	23 (22.5)	10 (23.8)	14 (23.3)	0.96
Perception of own high risk sexual behavior	16 (15.5)	8 (19.0)	8 (13.3)	0.44
Suspected sexual partners infidelity	14 (13.7)	1 (2.4)	13 (21.7)	0.01
Confirmatory test	10 (9.8)	5 (11.9)	4 (6.7)	0.36
Other reasons	7 (6.9)	1 (2.4)	6 (10.0)	0.14
**Total**	**102 (100%)**	**42 (100)**	**60 (100.0)**	**Not applicable**

### Quality and accessibility of VCT services

#### Training and Supervision of counselors

The counseling centers had between 3 and 5 counselors. Of the 26 counselors, 14 were lay volunteers, 7 health surveillance assistants and 5 nurses. No clinical officer, medical assistant or physician was a counselor at any of the sites. Only one of the counselors lacked specific training in HIV and AIDS counseling. 20 counselors reported that they were regularly supervised.

#### Availability of supplies and relevant infrastructure

All the facilities were using rapid HIV testing kits. Testing was being conducted on blood specimens. The clients waited between 15 to 20 minutes before being provided with test results. Three of the facilities had no waiting areas for clients and the same number had no lockable drawers. All the facilities had adequate gauze and/or cotton and antiseptics for cleaning blood collection sites. Different test kit brands were used at the different sites. Clinic records also showed that the clinics had used different brands of testing kits at different times. However all sites had test kits and other supplies on hand on the days of the study. All sites had male condoms to give out to clients and only 1 had female condoms. All the sites have 'penis models' for demonstration of correct male condom use and standardized data collection forms. At the time of the study, only one facility had an ARV treatment center on site. Five testing sites were linked to community youth support groups.

## Discussion

We have conducted an exploratory survey of HIV voluntary counseling and testing within community-based public health centers in Blantyre, Malawi. Voluntary counseling and testing facilities had supplies relevant to provision of testing services to clients. In terms of counselors, there was a mixture of health workers i.e. lay volunteers, nurses and health surveillance assistants providing counseling and testing services. The daily workload per counselor was low with each counselor was consulted by 5 to 12 clients in a 7 hour work day.

We also found that 74% of the study participants were less than 30 years old. We believe this could either be a reflection of the population structure of the country where the young predominates within population. Less than 30 year olds make up at least 75% of the population in Malawi [10]. It is also possible that the young adult groups and adolescents may feel more at risk due to their own sexual behaviors compared to older adults. It is also plausible to consider that perhaps younger individuals are more amenable to health messages than older persons. Our study did not collect data to support any of these suggestions though.

The primary motivations reported for accessing VCT services suggested that the majority of individuals had reasons to be concerned about their own sexual behaviors or behaviors of their sexual partners. Current illness was also an important factor in about 23% of the VCT clients. At least one third of the study participants reported that they presented for counseling and testing because of recent information on HIV and AIDS. Awareness of HIV, AIDS and testing in Malawi has been reported to exceed 98% among adults [2]. It is therefore of interest to note that many of the study participants still reported recent information as the trigger for testing. Although we did not explore what was actually meant by such responses, it is possible that it is a particular kind of message that may have appealed to the individual. This reason that new information could trigger desire to seek counseling was also identified as one of the primary reasons for HIV testing in Thyolo district, Malawi [9]. Klein et al reported on women who perceived themselves as being at zero risk of being HIV infected and yet when assessed by the study team, depending on their actual sexual history, would be classified as being at some risk of infection [11].

A study from rural Malawi reported that perception of high risk of HIV infection was associated with likelihood to accept HIV counseling and testing [12]. In a study from Zimbabwe, Morin et al reported that individuals who perceived themselves as being at high risk of HIV infection were more likely to present at mobile HIV testing sites [13]. Hutchinson and Mahlalela however, in a study from South Africa, reported that having multiple sexual partners within the past 12 months was not associated with increased HIV testing [14]. Another study by Daniel and Oladapo from Nigeria reported that pregnant women who perceived themselves to be at lower risk of being infected were more likely to accept HIV testing than those who thought they may be infected [15]. Thierman et al [16] reported in a study from Zambia, also found that women with low self-perceived risk of HIV infection were more likely to undergo testing. These studies suggest that motivations for HIV testing vary from place to place and could vary from time to time. It is therefore important that these motivations are studied periodically as it may not be appropriate to assume that the motivations from one study setting, or from another time period may be applicable in a setting.

While our study was not designed to obtain an answer to the likelihood of testing among a particular group, clients of VCT in our study reported that they had presented for testing because of ill-health, own perception of high risk sexual behavior or suspected partner infidelity.

In Malawi, it has been observed that women outnumber men as clients of highly active antiretroviral therapy programs [17]. It is currently not clearly known as to why women in southern Africa, women could be accessing HIV services more than their male counterparts [18]. However, although our study was not designed to assess the gender description of clients accessed VCT, it is interesting to note that when consecutive clients were recruited in this study, about 60% were females.

The linkage of HIV testing centers to community support groups such as youth groups has been reported as an important aspect that may promote continuity of care. Zachariah et al [19] have reported better clinical outcomes among HIV infected persons in living communities served by HIV-focused community support groups compared to community without such support groups. We found that only one testing facility was not connected to a community group.

Three of the sites had no lockable drawers. We believe this situation could compromise the security of supplies. However we did not feel patient confidentiality could be compromised as patients attending VCT in Malawi are not required to provide personal identifiers. Therefore in the event that an authorized person has accessed to the clinic records, there is very limited possibility for breach of confidentiality such that test results may be to any individual. Counseling and testing within the framework of medical consultation obviously will include personal identifiers.

## Limitations of the study

Our study has a number of limitations. Firstly, the sample size collected in this study is small, so the results may not be generalisable to the population seeking VCT services in Blantyre. As the sample included only those voluntarily accessing services, the results can also not be extrapolated to the general population.

The client data from which our analysis and interpretations are based were collected through self-report. To the extent that any of the study participants mis-reported, our results could be biased. As we did not observe the actual counseling sessions to assess time spent per client or the content and conduct of counseling session, our attempt to assess the quality of services provided is limited. However, we have documented that reagents and other supplies that will enable testing were available at the time of the interview. Compared to limited availability of essential medicines in the public health sector in Malawi [20,21], we find the availability of test kits at all the sites reassuring.

Furthermore, although we have also assessed the differences between males and females, our study had limited sample size. Future studies should attempt to answer whether the motivations in seeking HIV testing do indeed differ between males and females.

## Conclusion

HIV counseling and testing facilities were available in Blantyre city in six public health facilities. The majority of counseling and testing clients were motivated by perceptions of being at risk of HIV infection. While our study has added information to the literature on motivation for HIV testing in Malawi, there are a number of recommendations we would wish to suggest. Firstly we believe there is need for a future study with a larger sample size in order to validate our findings. We also suggest that future studies aimed at assessing the quality of HIV testing utilize clinic observation and exit interviews in order to comprehensively characterize care issues. We also suggest that public health policy makers target awareness intervention programs aimed at motivating individuals who may perceive themselves to be at lower risk to prevent for HIV counseling and testing services.

## Abbreviations

AIDS: acquired immunodeficiency syndrome

HIV: human immunodeficiency virus

VCT: voluntary counseling and testing

## Competing interests

The author(s) declare that they have no competing interests.

## Authors' contributions

JHB conceived the idea of the study, designed data collection tools, led data collection and analyzed the data. ASM participated in the design of the study, supervised data collection and participated in the analysis. All authors contributed to the writing of the manuscript. All authors read and approved the final manuscript.

## Pre-publication history

The pre-publication history for this paper can be accessed here:


